# High Risk of Severe Anaemia after Chlorproguanil-Dapsone+Artesunate Antimalarial Treatment in Patients with G6PD (A-) Deficiency

**DOI:** 10.1371/journal.pone.0004031

**Published:** 2008-12-29

**Authors:** Caterina I. Fanello, Corine Karema, Pamela Avellino, Germana Bancone, Aline Uwimana, Sue J. Lee, Umberto d'Alessandro, David Modiano

**Affiliations:** 1 Centre for Vaccinology and Tropical Medicine, Nuffield Department of Medicine, Oxford University, Oxford, United Kingdom; 2 National Malaria Control Program, Kigali, Rwanda; 3 Department of Public Health Sciences, University of Rome La Sapienza, Rome, Italy; 4 Mahidol-Oxford Tropical Research Unit, Faculty of Tropical Medicine, Mahidol University, Bangkok, Thailand; 5 Prince Leopold Institute of Tropical Medicine, Antwerp, Belgium; University of Cape Town, , South Africa

## Abstract

**Background:**

Glucose-6-phosphate dehydrogenase (G6PD) deficiency is the most common inherited human enzyme defect. This deficiency provides some protection from clinical malaria, but it can also cause haemolysis after administration of drugs with oxidant properties.

**Methods:**

The safety of chlorproguanil-dapsone+artesunate (CD+A) and amodiaquine+sulphadoxine-pyrimethamine (AQ+SP) for the treatment of uncomplicated *P. falciparum* malaria was evaluated according to G6PD deficiency in a secondary analysis of an open-label, randomized clinical trial [1]. 702 children, treated with CD+A or AQ+SP and followed for 28 days after treatment were genotyped for G6PD A- deficiency.

**Findings:**

In the first 4 days following CD+A treatment, mean haematocrit declined on average 1.94% (95% CI 1.54 to 2.33) and 1.05% per day (95% CI 0.95 to 1.15) respectively in patients with G6PD deficiency and normal patients; a mean reduction of 1.3% per day was observed among patients who received AQ+SP regardless of G6PD status (95% CI 1.25 to 1.45). Patients with G6PD deficiency recipients of CD+A had significantly lower haematocrit than the other groups until day 7 (p = 0.04). In total, 10 patients had severe post-treatment haemolysis requiring blood transfusion. Patients with G6PD deficiency showed a higher risk of severe anaemia following treatment with CD+A (RR = 10.2; 95% CI 1.8 to 59.3) or AQ+SP (RR = 5.6; 95% CI 1.0 to 32.7).

**Conclusions:**

CD+A showed a poor safety profile in individuals with G6PD deficiency most likely as a result of dapsone induced haemolysis. Screening for G6PD deficiency before drug administration of potentially pro-oxidants drugs, like dapsone-containing combinations, although seldom available, is necessary.

**Trial Registration:**

ClinicalTrials.gov NCT00461578

## Introduction

Glucose-6-phosphate dehydrogenase (G6PD) plays a critical role in protecting red cells from oxidant haemolysis. G6PD allows regeneration of NADPH, which is essential for glutathione recycling and protection against oxidative damage. G6PD deficiency is the most common inherited human enzyme defect, present in more than 400 million people worldwide. The gene encoding G6PD is located on the X chromosome; G6PD-deficient hemizygous males and homozygous females are vulnerable to oxidant haemolysis, whereas heterozygous females have variable (milder) deficiency determined by the degree of X-chromosomal inactivation.

There are numerous polymorphisms of the G6PD gene. Approximately 200 variant alleles have been described and 140 mutations, or combination of mutations, have been identified [2]. The high prevalence of G6PD deficiency in the tropics and Mediterranean areas is attributed to a protective effect against clinical *P. falciparum* malaria [3], [4]. Despite extensive heterogeneity, a single molecular variant, G6PD A- predominates in sub-Saharan Africa where it affects 15 to 20% of the African population, although there are marked differences between regions within the continent [5]. The A- variant differs from the normal G6PD B allele by two missense mutations, an A to G transition at position 376, encoding the so called “B” to “A” change (Asn126Asp), and a G to A transition at position 202, encoding the “A-” change (Val68Met). This additional mutation differentiates the “A-” allele with 12% enzymatic activity, from the “A” allele with 85% activity [6].

Acute haemolytic anaemia is the most frequent clinical manifestation associated with G6PD deficiency. This can be precipitated by foods or drugs with oxidant properties. The degree of drug-induced G6PD deficiency related haemolysis depends on a number of factors including the G6PD variant, the drug and dosage, and poorly characterised disease factors. Sulphonamides and sulphones are still widely used in tropical countries, and may cause haemolysis in patients with G6PD deficiency. Dapsone is used as a treatment of leprosy, some skin conditions, and more recently *Pneumocystic carinii* pneumonia [7]. It is used in combination with pyrimethamine, proguanil and chlorproguanil for malaria prophylaxis and malaria treatment. The most frequent adverse effects of dapsone are haematological. These include methaemoglobinaemia, haemolysis and anaemia, and less commonly agranulocytosis and idiosyncratic reactions (known as dapsone hypersensitivity syndrome). Patients with genetic deficiencies of enzymes involved in oxidant defences, such as G6PD or glutathione reductase, are more susceptible to haemolysis [7], [8].

In 2005–2006 an open label, randomised clinical trial was carried out in Rwanda to evaluate the safety and efficacy of artesunate+chlorproguanil-dapsone and amodiaquine+sulphadoxine-pyrimethamine for the treatment of uncomplicated *P. falciparum* malaria in children [1].

We evaluated the safety profiles of these two antimalarial treatments according to G6PD deficiency.

## Methods

### Study design

The drug trial has been described in detail elsewhere [1]. Briefly, we tested efficacy and safety of chlorproguanil-dapsone co-administered with artesunate (CD+A), compared to amodiaquine combined with sulphadoxine-pyrimethamine (AQ+SP), for the treatment of uncomplicated *P. falciparum* malaria in Rwanda. The trial was open label; 800 patients, aged 6–59 months, with uncomplicated malaria were randomised to receive AQ+SP (400) or CD+A (400). Patients were hospitalized for 4 days, so that appropriate management, including blood transfusion, could be performed and followed–up weekly until day 28 after treatment. Clinical and parasitological outcomes were recorded according to WHO guidelines [9]. Only patients with Packed Cell Volumes (PCV; haematocrit) ≥21% (corresponding to haemoglobin 7 g/dL) were included in the trial [10]. PCV was measured by microhaematocrit centrifugation each day during treatment and at each visit during follow-up. If necessary the measure was repeated twice a day. Blood was collected on Whatman filter paper for genotyping.

The initial protocol of the clinical study included an arm with CD alone, but this arm was discontinued because of the remarkably high number of failures observed at an early stage and thereafter patients were allocated to CD+A or AQ+SP only.

### Drug administration

CD+A: CD was administered orally at doses of 2.0 mg/kg chlorproguanil and 2.5 mg/kg dapsone once daily for 3 days using commercial Lapdap® paediatric tablets containing 15 mg of chlorproguanil hydrochloride and 18.75 mg of dapsone (GlaxoSmithKline, Brentford, UK) together with artesunate 4 mg/kg/day for 3 days using 50 mg tablets (Sanofi-Aventis, Gentilly Cedex, France).

AQ+SP: AQ was administered orally at a dose of 10 mg/kg daily for 3 days and SP was co-administered at a dose of 25 mg/kg sulphadoxine plus 1.25 mg/kg pyrimethamine the first day. AQ+SP and artesunate were both provided by Sanofi-Aventis (Gentilly-Cedex, France).

### Definitions

A patient was defined as an *Early Treatment Failure* (ETF) if she/he had any of the following: i) danger signs or severe malaria on days 1, 2 or 3 with parasitaemia; ii) parasite density at day 2 greater than at day 0; iii) parasitaemia on day 3 with axillary temperature≥37.5°C and iv) parasite density at day 3 equal to or greater than 25% of that at day 0 [9]. A *Serious Adverse Event* was defined as any untoward medical occurrence that at any dose results in death, requires inpatient hospitalisation or prolongation of existing hospitalisation, results in persistent or significant disability/incapacity or is life-threatening.

### Ethics

The clinical protocol of the original study was approved by the Ministry of Health of Rwanda, the ethical Committees of the London School of Hygiene and Tropical Medicine, London, UK, and the Prince Leopold Institute of Tropical Medicine, Antwerp, Belgium. As soon as the analyses of the blood samples were available, the families of children with G6PD deficiency were traced and informed by a medical doctor with a health worker. An explanatory health-card was given to the families which could be presented to hospital.

### G6PD (A-) genotyping

DNA extraction from Whatman filter papers was performed using standard Chelex method [11]. The G6PD A- allele (202G→A) has been characterized using the following primers: FOR 5′- CTG GCC AAG AAG AAG ATC TAC CC-3′ and REV 5′- GAG AAA ACG CAG CAG AGC ACA G 3′. DNA (50–100 ng) was amplified in a total reaction volume of 30 µl consisting of reaction buffer, 2 mM MgCl_2_, 0.1 µmol of each primer, 0.1 mM dNTPs each and 1 U of BioTaq DNA polymerase (Bioline). A touchdown program was used to prevent non-specific amplification; an initial denaturation step at 95°C for 5 min was followed by 14 cycles at 95°C for 40 s, 68.5°C for 40 s, and at 72°C for 40 s, with annealing temperature decreasing 0.5°C per cycle; the programme was completed by 23 additional cycles with annealing temperature 61°C and a final extension step at 72°C for 10 min. Amplified fragments were digested with NlaIII (NEBiolabs) restriction enzyme at 37°C for 4 hours and analysed with 2.5% MetaPhor (Cambrex) gel electrophoresis.

### Statistical analysis

Data were double-entered using Microsoft Access 2003 and validated using Epi Info 3.3.2 (CDC, Atlanta, GA, USA). All analyses were performed using STATA statistical analysis software package version 10.0 (StataCorp., College Station, TX, USA).

Patients were categorised into four groups for comparison: AQ+SP G6PD (B) (without G6PD deficiency), AQ+SP G6PD (A-) (with G6PD deficiency), CD+A G6PD (B) (without G6PD deficiency) and CD+A G6PD (A-) (with G6PD deficiency). Homozygous females and hemizygous males were considered as patients with G6PD deficiency. We also conducted a sensitivity analysis including the heterozygous G6PD females in the group of patients with G6PD deficiency.

Continuous data with a normal distribution were compared using ANOVA and the non-parametric Kruskal-Wallis test was used to analyse continuous data with a skewed distribution. Proportions were compared using chi-squared or Fisher's exact test.

The overall fractional reduction in haematocrit was defined as the difference between the patient's lowest level of haematocrit and that at baseline (i.e., pre-treatment) divided by the haematocrit at baseline. The fractional reduction from day 0 to day 3 was calculated in a similar manner.

The differences in the rate of decline in haematocrit levels from day 0 to day 3 were assessed in a separate model from the rate of recovery from day 7 to day 28. For each model, a regression line was fit for each patient and differences between groups as well as within group variation were adjusted for using random effects. The models were also adjusted for parasitaemia and temperature at presentation. The Odds Ratios (OR) and Risk Ratios (RR) were calculated with 95% Confidence Intervals (95% CI) using a 2-sided Fisher's exact test.

## Results

In the original study 792 children were studied of whom 702 were successfully genotyped for the G6PD variant A- (at position 202). For the remaining patients (10.3% in the AQ+SP arm and 14.2% in the CD+A arm; p = 0.1) samples were not received or DNA extraction was not successful. The total G6PD allelic frequency in the study population was 9.6% in the males (34 hemizygous subjects and 320 normal subjects) and 7.5% in the females (44 heterozygous and 4 homozygous females and 300 normal subjects), p = 0.3 (Table 1). The PCV at baseline (pre-treatment) was comparable between G6PD hemizygous and homozygous children (median PCV = 32%, range 23–39), G6PD heterozygous children (median PCV = 33%, range 25–38) and normal children (median PCV = 33%, range 21–46). Demographic and clinical characteristics pre-treatment were also similar in the four defined groups: AQ+SP G6PD (B), AQ+SP G6PD (A-), CD+A G6PD (B) and CD+A G6PD (A-) (all p>0.1; Table 2).

**Table 1:** G6PD (A-) frequency according to sex and treatment (AQ+SP: amodiaquine+sulphadoxine-pyrimethamine; CD+A chlorproguanil-dapsone+artesunate).

	CD+A	AQ+SP	Total
**Boys**			
Hemizygous	18	16	34
Normal	162	158	320
Total	180	174	354
G6PD (A-) % frequency	*10.0*	*9.2*	*9.6*
**Girls**			
Heterozygous	23	21	44
Homozygous	3	1	4
Normal	137	163	300
Total	163	185	348
G6PD (A-) % frequency a	*8.9*	*6.22*	*7.5*

a Comparison between boys and girls p = 0.3.

**Table 2:** Demographic and clinical characteristics of children at enrolment according to treatment (AQ+SP: amodiaquine+sulphadoxine-pyrimethamine; CD+A chlorproguanil-dapsone+artesunate) and presence of the G6PD (A-) deficiency allele (hemizygous males, homozygous and heterozygous females).

Demography and clinical characteristics	AQ+SP G6PD(B)	AQ+SP G6PD(A-)	CD+A G6PD(B)	CD+A G6PD(A-)
Female∶Male	163∶158	22∶16	137∶162	26∶18
Mean age in months (SD)	28.3 (14.1)	31.9 (14.3)	29.0 (14.9)	30.1 (14.0)
Mean weight in kg (SD)	11.0 (2.5)	11.8 (2.6)	11.2 (2.4)	11.0 ( 2.2)
Mean temperature °C (SD)	38.5 (1.3)	38.4 (1.5)	38.5 (1.3)	38.9 (1.2)
Axillary temperature ≥37.5°C (%)	230/321 (71.7)	26/38 (68.4)	221/299 (73.9)	35/44 (79.6)
Geometric mean asexual *P.falciparum*/*µ*L (95%CI)	22262.8 (19697.6–25162.0)	28158.0 (19348.4–40978.6)	22731.5 (19860.0–26018.2)	26808.5 (18950.4–37925.0)
Median PCV (range)	33 (22–43)	34 (23–39)	33 (21–46)	31 (23–38)
Splenomegaly (%)	2/321 (0.6)	0/38	4/295 (1.34)	0/44
Hepatomegaly (%)	0	0	0	0

### Haematocrit changes in relation to drug treatment and G6PD genotype with the heterozygous females considered as normal subjects

#### Univariate analysis

**Figure 1:**
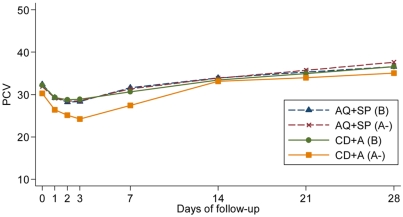
Mean PCV according to treatment group (AQ+SP: amodiaquine+sulphadoxine-pyrimethamine and CD+A: chlorproguanil-dapsone+artesunate), G6PD status (deficient: hemizygous males and homozygous females) and day of follow-up.

Between day 1 and 3 the mean PCV decreased in all four groups. After day 7, mean PCV increased steadily in all groups (Figure 1). The largest overall mean (SD) fractional reduction was observed in the CD+A G6PD (A-) group (Table 3). The proportion of patients whose PCV fell below 20 and 25% was also significantly higher in the CD+A G6PD (A-) group when compared with all other groups; when all other patients were combined into one group, the odds of a severe reduction in the haematocrit (defined as the proportion of patients whose PCV fell below 20%) was 9 times more likely to occur in patients with G6PD deficiency recipients of CD+A (OR = 9.2, 95% CI 2.8 to 30.2, p<0.001).

**Table 3:** Mean fractional reduction (±SD) in PCV according to treatment group (AQ+SP: amodiaquine+sulphadoxine-pyrimethamine; CD+A: chlorproguanil-dapsone+artesunate), G6PD status (deficient: hemizygous males and homozygous females) and proportion of patients whose PCV fell below 20 and 25%.

Group	n	Fractional reduction (SD)	No. with PCV below 20% (%)^b^	No. with PCV below 25% (%)
CD+A G6PD (B)	322	14.4 (7.9)	7 (2.2)	71 (22.0)
CD+A G6PD (A-)	21	22.3 (11.8)	4 (19.0)	10 (47.6)
AQ+SP G6PD (B)	342	16.4 (9.3)	9 (2.6)	93 (27.2)
AQ+SP G6PD (A-)	17	15.1 (9.4)	1 (5.9)	4 (23.5)
		p = 0.0001	p = 0.005	p = 0.05

b Results by gender: CD+A G6PD (B) 5 girls and 2 boys; CD+A G6PD, 4 boys; AQ+SP G6PD (B) 1 girl and 8 boys; AQ+SP G6PD (A-) 1 girl.

The mean fractional reduction (SD) from day 0 to day 3 was also highest in the CD+A G6PD (A-) group: 19.8% (12.5) compared with 12.0% (10.6) in the AQ+SP G6PD (B) group, 11.6% (9.34) in the AQ+SP G6PD (A-) group and 10.0% (9.26) in the CD+A G6PD (B) group (p<0.001).

#### Multivariate analysis

Between day 0 and day 3 compared with patients in the AQ+SP G6PD (B) group, those in the CD+A G6PD (A-) group had a significantly higher rate of decline in PCV, after adjustment for (log) parasitaemia and temperature at day 0 (p = 0.04); whereas PCV levels declined at the same rate in the AQ+SP G6PD (A-) group and CD+A G6PD (B) group when compared with patients in the AQ+SP G6PD (B) group (p = 0.79 and p = 0.56, respectively). In the first 4 days, haematocrit declined, on average, 1.3% per day for all patients who received AQ+SP treatment, regardless of G6PD status (95% CI 1.25 to 1.45). For patients in the CD+A G6PD (B) group, haematocrit levels declined, on average, 1.05% per day (95% CI 0.95 to 1.15) whereas in the CD+A G6PD (A-) group, the mean haematocrit decline was greater, 1.94% per day (95% CI 1.54 to 2.33).

From day seven, the haematocrit increased steadily in all groups. The rate of increase per day was similar (0.24%) for all patients treated with AQ+SP regardless of G6PD status (95% CI 0.22 to 0.25) and after adjustment for (log) parasitaemia at day 0. Haematocrit levels for patients in the CD+A (B) group increased 0.27% per day (95% CI 0.26 to 0.29) and 0.35% per day (95% CI 0.28 to 0.42) in patients in the CD+A G6PD (A-) group.

### Sensitivity analysis with heterozygous females considered as deficient subject

When we included the heterozygous females in the deficient groups the findings remained the same. At day one after treatment the haematocrit was lower than at baseline, but still comparable in the four groups (p = 0.12); after day 1 the reduction was again greater in patients in the CD+A G6PD (A-) group, which had a significantly lower mean PCV at days 2 and 3 (p = 0.01 and p<0.001 respectively). After day 7, results were identical.

In the multivariate analysis, those in the CD+A G6PD (A-) group had a significantly higher rate of decline in PCV compared with patients in the AQ+SP G6PD (B) group, (p = 0.004) after adjustment for (log) parasitaemia and temperature day 0; whereas PCV levels in the AQ+SP G6PD (A-) group and CD+A G6PD (B) group declined at the same rate when compared with patients in the AQ+SP normal group (p = 0.99 and p = 0.34 respectively). From day 7, results were also comparable: the rate of increase per day was similar for all patients treated with AQ+SP regardless of G6PD status (p = 0.28) and both CD+A G6PD (A-) and G6PD (B) patients had a higher increase compared to AQ+SP G6PD (B) patients (p<0.001) after adjustment for (log) parasitaemia at baseline.

### Blood transfusions

During the study 12 patients had a rapid fall of the haematocrit with other danger signs or severe malaria on days 1, 2 or 3 with parasitaemia (general danger signs of severe illness were defined as: inability to drink or breastfeed; vomiting; recent history of convulsions; lethargy or unconsciousness and inability to sit or stand up). Nine of those received a blood transfusion within four days of recruitment along with parenteral quinine and other supportive therapies (table 4). Four patients (3 G6PD hemizygous males and 1 non-deficient female) were treated with CD+A and 5 other patients (1 homozygous and 1 heterozygous female and 3 normal subjects males) that received a blood transfusion were treated with AQ+SP. Another patient, G6PD (B) treated with CD+A, required a blood transfusion at day 14. He was diagnosed with a concomitant disease and treated accordingly.

**Table 4:** Relative Risk (95% CI) of receiving a blood transfusion according to G6PD status following treatment with chlorproguanil-dapsone+artesunate (CD+A) or amodiaquine+sulphadoxine-pyrimethamine (AQ+SP).

	CD+A	AQ+SP	TOTAL
**No. receiving blood transfusion**			
Normal	2^c^/299	3/321	5/620
Heterozygous girls	0/23	1/21	1/44
Homozygous girls	0/3	1/1	1/4
Hemizygous boys	3/18	0/16	3/34
**Relative Risk**			
Normal	1	1	1
Hemizygous boys	24.9 (4.4, 139.8)	*Not Applicable*	10.9 (2.7, 43.9)
Hemizygous boys+homozygous girls	21.4 (3.8, 120.9)	6.3 (0.7, 57.4)	13.1 (3.7, 46.6)
Hemizygous boys+homozygous and heterozygous girls	10.2 (1.8, 59.3)	5.6 (1.0, 32.7)	7.6 (2.2, 25.6)

c One patient G6PD (B) was transfused at day 14 for a concomitant disease. Results excluding this case are: RR 49.8 (5.5, 455.5), RR 42.7 (4.6, 393.1) and RR 20.4 (2.2, 191.7), for hemizygous boys, hemizygous boys+homozygous girls, and hemizygous boys+homozygous girls+heterozygous girls, respectively.

Those cases were classified as Early Treatment Failures (apart from the patient who developed severe anaemia at day 14) with possible Serious Adverse Events as clinically it was not possible to distinguish whether the haemolysis was a consequence of the malaria infection only or also drug related. It is possible that the poor efficacy of AQ+SP, which resulted in a higher number of Early Treatment Failures compared to CD+A treatment [1], contributed to the need for a blood transfusion.

As mentioned in the methods, at the beginning of the trial 41 patients were allocated to CD alone; data from this arm were not included in the efficacy analysis [1] nor are they included in the present analysis. It is however worth mentioning that 2 patients in this group, a G6PD (A-) hemizygous male and a non-deficient male were transfused within 48 hrs.

Overall the risk of receiving a blood transfusion was about 7.6 times higher in G6PD (A-) patients, including heterozygous girls, (95% CI 2.2 to 25.6) compared to G6PD (B) patients regardless of the antimalarial treatment (Table 4). If we consider the risk in relation to the drug administered, G6PD (A-) individuals treated with CD+A had a Risk Ratio of 10.2 compared to G6PD (B) individuals (95% CI 1.8 to 59.3), whereas G6PD (A-) individuals treated with AQ+SP had a Risk Ratio of 5.6 (95% CI 1.0 to 32.7) compared with G6PD (B) individuals treated with the same drug.

### White Blood Cell analysis

The median WBC count was similar in the treatment groups at recruitment and at each day of the follow-up, 7, 14, 21 and 28 (all p>0.1; data not shown).

## Discussion

Malaria is a major cause of anaemia in tropical areas. In this study, conducted in a high transmission area, the haematocrit fell during acute malaria. This initial reduction was independent of the treatment received. Malaria causes anaemia through haemolysis of parasitized erythrocytes, accelerated clearance of uninfected erythrocytes, impaired compensation for this loss by bone marrow dyserythropoeisis and haemolysis due to oxidative stress [12]. Haemolysis may be exacerbated by some antimalarial drug treatment. In the present study, G6PD hemizygous and homozygous Rwandan children with uncomplicated *P. falciparum* malaria treated with CD+A experienced an estimated decline in haematocrit of nearly 2% per day (equivalent to about 0.7 g/dL) compared with 1% per day in patients who received AQ+SP, regardless of G6PD status.

The significantly steeper decline in haematocrit in G6PD-deficient children treated with CD+A is most likely to have resulted from the use of sulphone dapsone and its metabolite hydroxylamine. Artesunate may cause a temporary suppression of reticulocytosis which does not translate into anaemia, but would not explain the differential effect in G6PD-deficient subjects. Wootton et al., [13] tested CD alone or in combination with different doses of artesunate. Although the study was not powered to conduct a formal comparison of safety between treatments, the safety profile of CD, including the haematological effects, did not appear to be influenced by the artesunate dose. General use of dapsone is limited because of adverse haematological reactions, particularly in subjects with G6PD deficiency. The life span of erythrocytes is reduced when the drug is taken, to a degree related to the dose and length of exposure and is more evident in persons with G6PD deficiency [7], [8], [14], [15]. Although haematological toxicity is normally dose-dependent, even at low daily dosage can cause serious adverse events. For the treatment of leprosy dapsone has been used at 1–2 mg/kg per day in children, but there is limited experience (or not documented) in children under five and in leprosy patients with G6PD deficiency. In our study 2.3–4.7 mg/kg CD once daily for 3 days was associated with increased haemolysis and a greater risk of transfusion in children with G6PD deficiency.

By the end of the 28 day follow-up the haematocrit of most patients, with or without G6PD deficiency, recovered to normal levels. Drug induced haemolysis is self-limiting in individuals with G6PD deficiency because only older red cells are destroyed during drug challenge as these are the cells which are most enzyme deficient, whereas newly produced erythrocytes have nearly normal levels of G6PD which enables them to resist drug-induced destruction [8].

In total, 10 patients had severe post-treatment haemolysis requiring blood transfusion and the risk of receiving a blood transfusion was higher in subjects with G6PD deficiency recipients of CD+A.

In this trial we recruited children with a PCV≥21%; we hospitalized and carefully monitored them for the whole administration of the drug, transfusing blood when necessary. Under normal conditions these children would have received the treatment at the health centre or the drug would have been self-administered at home with the consequent risk of life threatening haemolysis.

In heterozygous females, the degree of deficiency is determined by the outcome of X-chromosomal inactivation and on average they have less severe clinical manifestations, although some develop severe acute haemolytic anaemia. In fact one heterozygous subject in this trial needed a blood transfusion.

In 2002, a randomised clinical trial in Kenya [16] found that the degree of haemoglobin decline between days 0 and 7 was greater in children treated with CD than with SP; 6.9% (13/188) of children receiving CD developed severe anaemia (haemoglobin levels of 5 g/dL or less) and exited the trial compared with 1.5% (3/195) of children receiving SP. However no further analyses were performed on the G6PD deficiency status of the children.

In 2004, results of a randomised multicentre trial in Africa [10], [17] showed that patients with G6PD deficiency were at greater risk for anaemia after CD treatment (OR = 2.5; p<0.001) than after SP (OR = 0.9; p = 0.94), whereas there were no differences between patients without G6PD deficiency who were recipients of CD or SP (OR = 0.9; p = 0.62) . The risk for having a >4 g/dl drop in haemoglobin concentration was at least doubled in patients with G6PD deficiency after CD treatment (OR = 3.2; p = 0.02).

More recently, preliminary results of a multi-centre, double-blind Phase III trial of CD co-formulated with artesunate vs. artemether-lumefantrine [18], showed a significant reduction in haemoglobin due to haemolytic anaemia in patients with G6PD deficiency, with lowest levels of haemoglobin occurring seven days after treatment. Fifteen patients, all in the CDA group and 13 of whom with G6PD, had severe post-treatment haemolysis requiring blood transfusion in the study.

The prevalence of G6PD deficiency in African populations varies from 28.1% in southwest Nigeria to 22.5% in Congo, 15.7% in Mali, 13.0% in Uganda and 9.0–15.5% in Gabon [5]. In our study population the allele frequency of the G6PD was about 10% in boys and 8% in girls. In a previous study conducted in Rwanda [19] screening for G6PD deficiency using cord blood samples showed a phenotypic frequency of 3.8%.

Pro-oxidant drugs, including antimalarial drugs associated with haemolysis in G6PD-deficient individuals are used commonly in malaria endemic areas. Screening for G6PD deficiency before drug administration of potentially pro-oxidants drugs although seldom available, is necessary.

### Conclusions

Chlorproguanil-dapsone+artesunate in Rwanda had a poor safety profile in G6PD deficient individuals most likely as a result of dapsone induced haemolysis. This drug combination has also been previously reported to have poor efficacy (70% Adequate Clinical and Parasitological Response at day 28 PCR corrected [1]). CD is a fixed-dose combination produced by GlaxoSmithKline under the brand name of Lapdap™. The co-formulated combination with artesunate, Lapdap-plus™, was under development, but because it was associated with an increased risk of anaemia GSK has stopped the further development of the combination therapy and commenced a product recall process at pharmacy level in Kenya for Lapdap™ the only market with recent sales of the product [20].
